# Outcomes of an inpatient refeeding protocol in youth with Anorexia Nervosa and atypical Anorexia Nervosa at Children’s Hospitals and Clinics of Minnesota

**DOI:** 10.1186/s40337-016-0124-0

**Published:** 2016-12-19

**Authors:** Kathryn Smith, Julie Lesser, Beth Brandenburg, Andrew Lesser, Jessica Cici, Robert Juenneman, Amy Beadle, Sarah Eckhardt, Elin Lantz, James Lock, Daniel Le Grange

**Affiliations:** 1 Neuropsychiatric Research Institute, Fargo, North Dakota USA; 2 Children’s Hospitals and Clinics of Minnesota, Minneapolis, Minnesota USA; 3 The Children’s Hospital of Philadelphia, Philadelphia, Pennsylvania USA; 4 Fairview Health Services, Minneapolis, Minnesota USA; 5 Drexel University, Philadelphia, Pennsylvania USA; 6 Department of Psychiatry, Stanford University School of Medicine, Stanford, California USA; 7 University of California, San Francisco Department of Psychiatry, San Francisco, California USA

**Keywords:** Anorexia Nervosa, Refeeding, Hypophosphatemia, Children, Adolescents

## Abstract

**Background:**

Historically, inpatient protocols have adopted relatively conservative approaches to refeeding in Anorexia Nervosa (AN) in order to reduce the risk of refeeding syndrome, a potentially fatal constellation of symptoms. However, increasing evidence suggests that patients with AN can tolerate higher caloric prescriptions during treatment, which may result in prevention of initial weight loss, shorter hospital stays, and less exposure to the effects of severe malnutrition. Therefore the present study sought to examine the effectiveness of a more accelerated refeeding protocol in an inpatient AN and atypical AN sample.

**Methods:**

Participants were youth (ages 10–22) with AN (*n* = 113) and atypical AN (*n* = 16) who were hospitalized for medical stabilization. A retrospective chart review was conducted to assess changes in calories, weight status (percentage of median BMI, %mBMI), and indicators of refeeding syndrome, specifically hypophosphatemia, during hospitalization. Weight was assessed again approximately 4 weeks after discharge.

**Results:**

No cases of refeeding syndrome were observed, though 47.3 % of participants evidenced hypophosphatemia during treatment. Phosphorous levels were monitored in all participants, and 77.5 % were prescribed supplemental phosphorous at the time of discharge. Higher rates of caloric changes were predictive of greater changes in %mBMI during hospitalization. Rates of caloric and weight change were not related to an increased likelihood of re-admission.

**Conclusions:**

Results suggest that a more accelerated approach to inpatient refeeding in youth with AN and atypical AN can be safely implemented and is not associated with refeeding syndrome, provided there is close monitoring and correction of electrolytes. These findings suggest that this approach has the potential to decrease length of stay and burden associated with inpatient hospitalization, while supporting continued progress after hospitalization.

## Plain English Summary

Anorexia Nervosa (AN) has been shown to be the deadliest mental disorder due to the serious medical and psychiatric problems that are associated with this illness. Many hospital treatment programs start patients with AN on low calorie diets and increase their meal plans gradually. It has been thought that this approach reduces the chance of patients having refeeding syndrome, a potentially fatal complication that can happen when severely underweight individuals begin to eat more. However, a gradual approach to refeeding prolongs hospitalization and causes more disruption for patients and families. Our study examined a refeeding program that started patients with AN and atypical AN on higher calorie meal plans and advanced their calories more rapidly than traditional approaches to inpatient hospital treatment. Results showed that this protocol was related to increased weight gain, but was not related to re-admission or refeeding syndrome. Our findings support a more rapid approach to refeeding patients with AN and atypical AN in the hospital.

## Background

Children and adolescents with eating disorders may require inpatient hospitalization for medical stabilization and weight regain [1–3], with Anorexia Nervosa (AN) accounting for a significant proportion of such admissions [4]. Higher rates of weight gain and higher weight status upon discharge are generally predictive of better outcomes and weight maintenance after inpatient treatment [5–7]. Despite the importance of early weight changes in long-term recovery from AN, current national standards in the U.S. recommend relatively conservative approaches to refeeding in AN (e.g., starting at 1200 calories and increasing by 200 calories every other day) [8, 9].

Such “start low, go slow” approaches were developed with the aim of reducing the risk of refeeding syndrome, a potentially fatal condition that may occur when nutrition is re-introduced to a severely malnourished individual. Although there are no strict definitions, refeeding syndrome is characterized by a constellation of symptoms stemming from shifts in electrolytes that may occur when refeeding malnourished individuals, which can lead to potentially lethal complications [10–12]. Early signs of refeeding syndrome include low levels of serum phosphorous, magnesium or potassium. Refeeding syndrome may cause serious clinical complications including signs of fluid shifts (e.g., edema), respiratory distress, congestive heart failure, cardiac arrhythmias, and seizures [13]. This condition is a serious risk during refeeding and has been documented in reports of AN treatment [14].

Hypophosphatemia has emerged as a clear marker of risk of refeeding syndrome, and the central role of phosphorous during refeeding is well-described [15]. The risk of refeeding hypophosphatemia also appears to be greatest for patient’s presenting with more severe malnutrition [15, 16]. Per the recent review by Garber and colleagues [16], replacing phosphorus has not been associated with adverse side effects. Despite the general consensus and recommendation to monitor and replace phosphorous during nutritional rehabilitation of malnourished patients, there are not clear guidelines for how to replace phosphorous, especially with more accelerated refeeding protocols. At our center, hospitalists replace phosphorous when it is declining, thereby possibly avoiding hypophosphatemia and preventing refeeding syndrome.

Although the “start low, go slow” approach to refeeding attempts to minimize risk of refeeding syndrome, this approach also has significant downsides, in that it incurs more cost and burden to families, and in some studies has been shown to result in initial weight loss and slower rates of weight gain while during hospitalization [17]. These findings are particularly important in light of altered metabolic processes that have been documented in AN. That is, while individuals with AN demonstrate reduced metabolic rate [18], evidence suggests that during the refeeding process they become energy inefficient due to hypermetabolism and increased dietary-induced thermogenesis (i.e., energy production caused by metabolizing food consumed) [19–21], which may persist following weight restoration [2]. This is also evidenced by the fact that individuals with AN require more energy per kilogram of body weight than would be expected for normal weight individuals to gain weight [20, 22]. Therefore it is necessary to develop safe, evidence-based refeeding protocols that account for this phenomenon and provide adequate energy intake levels to accomplish ample weight regain early in treatment.

Furthermore, there is emerging evidence to suggest that hospitalized individuals with AN can tolerate more aggressive refeeding protocols, which is contrary to “start low, go slow” approaches [16, 23]. This is consistent with recently published Australian and New Zealand guidelines recommending a less conservative refeeding approach in conjunction with appropriate monitoring and supplementation of phosphorus [24]. Indeed, some studies of adolescents with AN have shown that higher calorie prescriptions and more rapid weight gain are not related to hypophosphatemia or other signs of refeeding syndrome [25–30]. Moreover, higher calories prescribed upon admission have been found to relate to shorter lengths of stay [17, 25, 26], which has the potential to increase the cost-effectiveness of treatment and reduce burden and disruption associated with hospitalization.

Evidence also suggests that higher initial calories predict higher rates of weight gain [25], yet some research has not replicated this finding [26]. In addition, it is not clear whether higher rates of weight gain are necessarily beneficial for patients’ long-term outcomes. While one study of adolescent and adult inpatients treated for AN found that higher rates of weight restoration were predictive of higher weight at 1-year follow-up [7], in another inpatient sample, more rapid weight gain was found to predict higher rates of re-hospitalization [31]. Additionally, in adolescents, some studies have found that weight status at admission and discharge was not related to clinical outcomes [32, 33].

Despite some variations between studies, a previous synthesis of studies of more rapid refeeding approaches to refeeding in AN summarized the evidence for the “start high, advance fast” approach to refeeding in AN patients (at 75–85 % of median Body Mass Index, %mBMI) in hospital as (1) beginning at 1500 kcal or higher; (2) advancing by 250 kcal every day or every other day to reach 2500–3000 kcal by day 14; (3) weekly weight gain of approximately 1.5 kg (3.3 lbs); (4) a lack of indicators of refeeding syndrome; and (5) achieving medical stability by approximately day 14 [34]. However, a recent review of refeeding protocols by Garber and colleagues [16] revealed that only seven studies thus far have examined higher calorie meal-based protocols, leaving much unknown about specific factors that influence patients’ weight gain during hospital stay and their continued weight restoration post-discharge. Furthermore, thus far only one RCT has compared different approaches to refeeding [35]. This study, which assessed young adults with a duration of illness of 3–4 years who had been previously hospitalized, compared a protocol consisting of nasogastric tube feedings plus meals to meals alone [35]. Thus, there have been no RCTs comparing different meal-based approaches to refeeding in children and adolescents during their first hospitalization. In the absence of such RCTs, retrospective studies have the potential to provide further insight into the effectiveness and acceptability of more rapid refeeding protocols.

Therefore, the present study sought to examine the safety and effectiveness of a more accelerated meal-based refeeding protocol for youth with AN and atypical AN who were treated on an inpatient unit. It was predicted that (1) this protocol would result in significant increases in weight status (%mBMI) without the occurrence of refeeding syndrome; (2) higher rates of caloric change throughout treatment would be related to greater change in %mBMI during and after hospitalization; (3) higher rates of caloric and %mBMI change during hospital stay would not be related to an increased likelihood of re-hospitalization; and (4) higher calories prescribed upon discharge would be predictive of greater %mBMI increase between the time of discharge and follow-up assessment.

## Methods

### Study design and participants

A retrospective chart review was conducted for consecutive admissions of patients (ages 22 and younger) with a primary diagnosis of AN or atypical AN^1^ who were treated in the inpatient medical stabilization unit for eating disorders at Children’s Hospitals and Clinics of Minnesota from 2012 to 2015. Only first episodes of inpatient hospitalization at this facility were included in the analyses. Admission and discharge criteria are described in Table 1. Admissions were based on clinical evaluation and the requirement to meet at least one admission criteria, which were based on previously established guidelines in the U.S.[1, 3]. Readiness for discharge was assessed by a team of hospitalists and eating disorder specialists, including psychiatrists, psychologists, and social workers. Patients were deemed ready for discharge when they showed improvement in vital signs and electrolytes, had adequate oral intake for weight regain, reached at least 75 % mBMI, had completed at least one therapist-led family meal and one family-led practice meal, and had an outpatient treatment plan in place. Participants who started outpatient treatment after discharge completed a follow-up assessment approximately 4 weeks after discharge. Data was not available for participants who did not follow-up in our facility. Participants were excluded from analyses if they received nasojejunal (NJ) or nasogastric (NG) tube feeding during treatment, as they did not follow the same refeeding protocol. No participants were excluded due to co-occurring medical or psychiatric conditions. This study was approved by the Institutional Review Board at Children’s Hospitals and Clinics of Minnesota.

**Table 1:** Inpatient admission and discharge criteria

Admission criteria	Discharge criteria
Weight <75 % of median BMI (%mBMI)	Medical stabilization
Rapid weight loss, even if weight not <75 %	Admission criteria are no longer met
Acute food refusal	Adequate oral intake for weight regain
Heart rate <50 bpm	Cessation of vomiting
Orthostatic hypotension (increase in pulse of >20 bpm or drop in blood pressure of 10–20 mmHg/min from supine to standing)	Has completed 1 staff led practice meal and 1–3 family led/individual practice meals
Blood pressure <80/50	
Hypokalemia	
Hypophosphatemia	
Hypomagnesemia	
Dehydration	
Cardiac arrhythmia	
Hypothermia	
Syncope	
Symptomatic hypoglycemia	
Lack of improvement/worsening symptoms despite outpatient treatment	
Uncontrolled vomiting or hematemesis	

### Refeeding protocol

The refeeding protocol consisted of three meals and two snacks (afternoon and evening). Meals and snacks were consumed in a supervised group dining room on the unit. If participants were unable to consume the food provided on their tray, they were offered a meal replacement (i.e., high-calorie liquid supplement) that provided an equivalent amount of energy. Participants were supervised by program staff for 60 min after meals and 30 min after snacks. In addition to meals, participants attended three groups per week on topics including emotional regulation, problem solving and interpersonal effectiveness/communication. Participants received additional services including music therapy, child life programming, physical therapy, and integrative medicine. Participants were typically on telemetry for up to the first 72 h following admission, or longer until their heart rate trends were approaching 50 beats per minute. While on telemetry, participants were required to remain on the unit. Following this, if participants were finishing meals they were allowed privileges such as going off the unit (within the hospital grounds) with parents or hospital staff. On the medical unit, bathrooms were not locked and participants were not given additional replacements if they were found to be engaging in exercise. Participants were under staff supervision for the majority of the day and into the evening, with additional support requested from parents if needed. Participants were required to be completing most meals and replacements to attend physical therapy groups, which occurred several times per week.

Most participants started on a 1500 kcal per day regimen. Calorie increases were made in 500 kcal increments in order to attain an inpatient rate of weight regain of 130–200 g per day. The program included collaborative weighing sessions twice weekly, in which progress with weight regain was discussed openly with the patient and family. The macronutrient composition of the diet consisted of 50–60 % carbohydrate, 20–30 % fat, and 15–20 % protein. Fluid requirements were calculated for each participant based on his/her current weight using the Holliday-Segar Method [36]. Intravenous (IV) fluids were administered if participants exhibited significant signs of dehydration, hypotension, and/or uncontrolled vomiting, or as otherwise clinically indicated. Daily fluid intake and urine output were recorded throughout hospitalization. Standard laboratory values were taken upon admission, and most were repeated at discharge. Electrolytes, including phosphorus, were checked at least twice weekly. While there ﻿are﻿ few data on inpatient supplementation protocols during refeeding for patients with AN [37], given our accelerated meal-based refeeding protocol, phosphorus was replaced frequently with the goal of maintaining a level of 4.0 mg/dL. Two formulations of phosphorus were utilized, including Neutra-Phos tablets and K-Phos Neutral tablets or packets.

### Data collection

Chart reviews were conducted to extract assessments of vital signs, laboratory values, and weight status during hospitalization. Follow-up weight status was assessed at participants’ 4-week follow-up in the outpatient clinic, where Family-Based Treatment (FBT) [38] or Cognitive Behavioral Therapy-Enhanced (CBT-E) [39] were offered as first-line treatments. Re-admission to the inpatient unit within 4 weeks of discharge was documented. While it was not possible to verify whether patients were admitted to another facility, Children’s is the only hospital in the region specializing in the medical stabilization of pediatric patients with eating disorders.

### Indicators of refeeding syndrome

Given the difficulties of quantifying all of the symptoms that comprise refeeding syndrome, it has been recommended that refeeding hypophosphatemia (i.e., serum phosphorus level below 3 mg/dL) be considered as an indicator of risk of refeeding syndrome [15]. Consistent with this guideline, this study evaluated risk of refeeding syndrome by assessing refeeding hypophosphatemia at any time during hospitalization. Chart reviews were also conducted for all participants to assess for the presence of full refeeding syndrome. In addition, hospitalists working in this unit consider the occurrence of multiple electrolyte imbalances during refeeding, specifically hypophosphatemia, hypokalemia, and hypomagnesemia, to be indicative of a high likelihood of full refeeding syndrome. Given this practice, in order to quantitatively operationalize a high likelihood of full refeeding syndrome, we also evaluated whether any participants evidenced all three of these electrolyte abnormalities during their treatment (but not necessarily concurrently).

### Statistical methods

Weight status was calculated based on the participant’s age, height, sex, and corresponding Centers for Disease Control and Prevention (CDC) 50th percentile BMI-for-age [40], which is the expected median BMI among normally developing adolescents of similar age, sex, and height. Participant’s weight status was expressed as a percentage of this value (%mBMI). The Estimated Energy Requirement (EER) of each participant was calculated retrospectively [41], which approximates the energy intake required to maintain weight in a healthy individual of similar age, sex, weight, and height at a low activity level (Daily PAL: 1.0). Given that the EER underestimates the energy needs of those with AN, EER was calculated based on the BMI corresponding to the CDC median BMI for age and sex, which is in line with previous research [25].

Caloric changes were calculated both as raw values and as percentages of participants’ EER in order to assess the degree of energy surplus that was provided relative to their EER. The rate of caloric change during treatment was calculated by dividing the change in calories between admission and discharge by their length of stay, expressed in kcal/day.

Growth curve models were used to assess the nature of change in %mBMI over time, and predictors of %mBMI change were subsequently added to these models using Hierarchical Linear Modeling (HLM) [42]. First, unconditional growth curve models investigated whether there was a linear or nonlinear change over time, which included both linear (*π*_*1i*_) and quadratic (*π*_*2i*_) growth parameters, e.g.:

**Level**-**1 Model**

$$ \%mBM{I}_{ti}={\pi}_{0i} + {\pi}_{1i}*\left(Tim{e}_{ti}\right) + {\pi}_{2i}*{\left(Tim{e}_{ti}\right)}^2 + {e}_{ti} $$

**Level**-**2 Model**

$$ {\pi}_{0i}={\beta}_{0 0}+{r}_{0i} $$

$$ {\pi}_{1i}={\beta}_{1 0}+{r}_{1i} $$

$$ {\pi}_{2i}={\beta}_{2 0}+{r}_{2i} $$

Given that it is likely that the rate of weight gain varied between the time of hospitalization and post-hospitalization, a piecewise linear growth model also estimated growth rates separately during hospitalization (admission to discharge) and post-hospitalization (discharge to follow-up). Time-varying (level 1) and person-level (level 2) predictors were subsequently added to models to assess the relationship between the rate of caloric change and %mBMI change during and after hospitalization. In these conditional models, the rate of caloric change was entered at level 1, while age and EER were entered at level 2. In the model assessing post-hospitalization %mBMI, discharge calorie level was also entered as a predictor. A Bernoulli model assessed whether higher rates of caloric and %mBMI change were related to an increased likelihood of re-hospitalization after discharge (binary outcome).

To evaluate the safety of the refeeding protocol, the number and percentage of participants who evidenced refeeding hypophosphatemia was assessed, as well as whether any cases met the aforementioned criteria for refeeding syndrome. A binary logistic regression model also assessed predictors (i.e., admission %mBMI, age, and rates of %mBMI and caloric changes) of hypophosphatemia at any point during treatment. Analyses were conducted using SPSS 24 [43] and HLM 7.01 [44].

## Results

### Sample characteristics

Table 2 displays characteristics of the sample. Eleven participants were excluded from analyses because they had NG/NJ tubes at some point during their treatment, which resulted in a total sample of 129. Participants were predominately female (94.6 %) and ranged in age from 10 to 22 (*M* = 15.84, *SD* = 2.37). Most identified as non-Hispanic Caucasian (89.1 %), and the remaining as Asian American (3.9 %), African American (1.6 %), Hispanic/Latino (1.6 %), American Indian/Alaskan 0.8 %), and multiracial (0.8 %). Three participants (2.3 %) did not report this information. There were five participants for which admission %mBMI could not be calculated because their age exceeded the maximum age specified in CDC BMI-for-age charts. These participants were excluded from analyses involving %mBMI estimations. Of the total sample, 113 (87.6 %) patients had AN, and 16 (12.4 %) had atypical AN.

**Table 2:** Descriptive statistics

	*N*	Min	Max	*M*	*SD*
Length of stay (days)	129	0.88	40.01	14.98	7.51
Participant age	129	10.04	22.94	15.84	2.37
Admission initial calories	129	1500.00	3000.00	1585.27	280.37
Day 14 prescribed calories	71	1500.00	5000.00	3626.17	644.68
Discharge calories	129	1500.00	5000.00	3771.32	829.17
Admission initial calories % of EER	129	29.02	131.32	64.52	14.34
Discharge calories % of EER	129	59.00	230.08	152.81	37.43
Admission to discharge calorie change	129	0.00	3500.00	2186.05	875.16
Calorie change (admission to discharge) % of EER	129	0.00	238.61	130.21	52.46
Admission %mBMI	124	61.51	110.64	79.40	8.47
Discharge %mBMI	124	64.40	111.18	85.54	8.11
Four-week follow-up %mBMI	74	72.07	113.70	89.84	7.92
Admission BMI	129	11.90	20.60	15.86	1.81
Discharge BMI	129	13.70	21.40	17.12	1.62
Days between discharge and follow-up	83	21.00	44.00	26.07	5.59
Days on telemetry	127	0.00	14.00	2.84	2.18
Days administered (IV) fluids	11	1.00	2.00	1.18	0.40
Fluid balance day 1(mL)	127	−4070.00	2818.00	−431.59	945.97
Fluid balance day 2 (mL)	126	−6005.00	1570.00	−733.80	1079.87
Fluid balance day 3 (mL)	122	−5125.00	1920.00	−480.36	1015.64
Rate of calorie change (kcal/day)					
Admission to discharge	129	0.00	539.12	163.87	72.65
Week 1 (Day 1–7)	116	−14.29	457.14	185.16	82.57
Week 2 (Day 7–14)	70	−123.81	300.00	120.48	76.66
Week 3 (Day 14–21)	28	−250.00	200.00	1.33	113.07
Week 4 (Day 21–28)	9	−100.00	392.86	80.95	136.59

%*mBMI* Percentage of median BMI, *EER* Estimated Energy Requirement, *BMI* Body Mass Index

%mBMI was not calculated for individuals over 20 years of age (*n* = 6) due to upper limits of the Center for Disease Control (CDC) BMI-for-age percentile charts

Fluid balance was calculated as total input minus total output

Of the 129 participants, 12 (9.3 %) were re-admitted within 4 weeks, and 89 (69.0 %) completed the 4-week follow-up. Using an adjusted significance level for multiple comparisons (*p* < .01), participants who did and did not complete follow up did not evidence significant differences in admission %mBMI (*t*[122] = 1.41, *p* = .162), discharge %mBMI (*t*[122] = 1.37, *p* = .175), length of stay (*t*[127] = .511, *p* = .610), rate of caloric change (*t*[127] = 1.80, *p* = .074), age (*t*[127] = 2.11, *p* = .036), or proportion of males compared to females (*χ* ^2^ [1] =1.97, *p* = .160).

### Safety and effectiveness

Laboratory values upon admission, during treatment, and at discharge are displayed in Table 3. Subsequent to admission, rates of hypophosphatemia, hypocalcemia, hyponatremia, hypokalemia, and hypomagnesaemia were 47.3 % (*n* = 61), 43.4 % (*n* = 56), 18.6 % (*n* = 24), 12.4 % (*n* = 16), and 1.6 % (*n* = 2), respectively. a Participant age, sex, rates of caloric or %mBMI change, or admission %mBMI were not significant predictors of hypophosphatemia during hospitalization (adjusting for length of stay). No participants evidenced hypophosphatemia at discharge, although 100 patients (77.5 %) were prescribed phosphorus supplements at discharge. Of the patients for whom the discharge phosphorus dose was available (*n* = 98), the mean dose was 1012.76 mg (*SD* = 611.71; range: 250–3000 mg). Despite the significant number of participants evidencing risk for refeeding syndrome during treatment, both chart review and quantitative assessment of refeeding criteria (i.e. hypophosphatemia, hypokalemia, and hypomagnesemia, each at some point during hospitalization) did not identify any cases of full refeeding syndrome.

**Table 3:** Laboratory values at admission, during treatment, and discharge

		Admission							During Treatment				Discharge						
					Below reference range		Above reference range		Below reference range		Above reference range					Below reference range		Above reference range	
Test	Reference range	*N*	*M*	*SD*	*N*	%	*N*	%	*N*	%	*N*	%	*N*	*M*	*SD*	*N*	%	*N*	%
Sodium	137–147 mEq/L	129	139.39	2.49	15	11.63	0	0	24	18.60	1	0.78	126	140.21	1.77	0	0	0	0
BUN	Age < 18: 9–18 mg/dL	129	14.57	5.62	13	10.08	24	18.60	28	21.71	40	31.01	126	14.36	4.13	7	5.43	14	10.85
	Age >18: 8–22 mg/dL																		
Potassium	3.5–5.3 mEq/L	129	3.87	0.37	11	8.53	0	0	16	12.40	2	1.55	126	4.15	0.30	0	0	0	0
Glucose	70–100 mg/dL	129	81.62	18.64	34	26.36	20	15.50	54	41.86	11	8.53	126	82.11	9.60	10	7.75	5	3.88
Calcium	8.6–10.6 mg/dL	129	6.65	2.30	69	53.49	0	0	56	43.41	0	0.00	126	6.61	2.28	72	55.81	0	0
Phosphorus	3.0–5.5 mg/dL	129	3.63	0.58	13	10.07	0	0	61	47.28	9	6.98	125	4.34	0.57	0	0	3	2.33
Magnesium	1.5–2.5 mg/dL	126	1.87	0.23	2	1.55	0	0	2	1.55	1	0.78	126	1.84	0.23	1	0.78	0	0
Chloride	98–106 mEq/L	129	103.84	3.16	5	3.88	24	19	6	4.65	74	57.36	126	105.10	2.46	0	0	31	24.03
Creatinine	0.38–0.99 mg/dL	129	0.77	0.16	0	0	15	11.63	0	0	14	10.85	125	0.68	0.14	0	0	4	3.10
AST ^a^	10–41 U/L	110	27.70	40.37	1	0.78	9	6.98											
ALT ^a^	30–65 U/L	110	43.32	68.15	45	34.88	9	6.98											
Albumin ^a^	3.5–5.0 g/dL	119	4.30	0.46	4	3.10	5	3.88											
Protein ^a^	6.0–8.0 g/dL	110	7.43	0.61	1	0.78	15	11.63											
WBC ^a^	4.5–13 K/UL	115	5.45	1.54	32	24.81	0	0											
TSH ^a^	.5–4.8 ulU/mL	103	1.81	1.49	9	6.98	5	3.88											
Prealbumin ^a^	21.2–42.4 mg/dL	37	22.08	5.36	17	13.18	0	0											

^a^ Not repeated during treatment or at discharge

Other medications prescribed to patients are shown in Table 4. With respect to psychotropic medications, a substantial proportion of patients (*n* = 57; 44.2 %) were prescribed antidepressants (i.e., fluoxetine, amitriptyline, citalopram, escitalopram), while a smaller number were prescribed atypical antipsychotic medication (*n* = 6; 4.7 %).

**Table 4:** Summary of medications prescribed to patients

Medication	*n*	%
Hydroxyzine	64	49.61
Polyethylene glycol	62	48.06
Fluoxetine	53	41.09
Ondansetron	22	17.05
Lansoprazole	15	11.63
Olanzapine	6	4.65
Amitriptyline	2	1.55
Citalopram	1	0.78
Erythromycin	1	0.78
Escitalopram	1	0.78

### Changes in calories

The majority of patients (89.9 %) were started on 1500 kcal meal plans upon admission, and on average, participants increased to over 3600 kcal by day 14. The mean prescribed calorie level at discharge (i.e., approximately 3771 kcal) represented over 150 % of participants’ EER (Table 2). As shown in Table 2, there was a mean increase of 163.87 kcal per day.

### Changes in weight and %mBMI

**Fig. 1:**
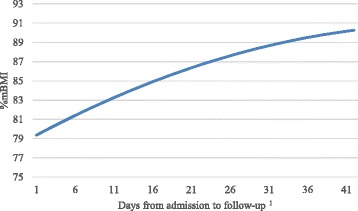
Displays the curvilinear change in %mBMI between admission and follow-up. ^1^The mean length of stay (14.98 days) and mean days between discharge and follow-up (26.07 days) were summed to determine the range of values for the x-axis. Follow-up data points represented only patients who completed this assessment

Participants evidenced a mean weekly weight gain of 1.39 kg (*SD* = 1.49). Table 5 displays results of growth curve analyses estimating changes in %mBMI. As displayed in Fig. 1, results indicated an average linear increase of 0.43 in %mBMI per day from the time of admission to follow-up, with the quadratic slope estimate indicating a significant deceleration over time (Table 5, Model 1). However, when considering only the time during hospitalization, there was not significant acceleration or deceleration in growth (Model 2), suggesting a linear change during hospitalization.

**Table 5:** Summary of growth curve model results

Dependent variable	Independent variables	Estimate	*SE*	*t*	*p*
1. %mBMI change	Intercept	79.36	0.78	101.17	<0.001
from admission	Time	0.43	0.02	17.43	<0.001
to follow-up	Time^2^	<0.01	<0.01	−7.55	<0.001
2. %mBMI change	Intercept	79.39	0.75	105.18	<0.001
during	Hospital time	0.37	0.09	4.38	<0.001
hospitalization	Hospital time^2^	<0.01	<0.01	0.59	0.558
3. %mBMI change during	Intercept	79.40	0.79	100.48	<0.001
hospitalization vs.	Hospitalization time	0.38	0.02	17.85	<0.001
post-hospitalization	Post-hospitalization time	0.09	0.01	6.56	<0.001
Conditional models					
4. %mBMI change	Intercept	79.28	1.02	77.66	<0.001
during	Age	−0.99	0.36	−2.72	0.007
hospitalization	Calorie rate	0.04	0.01	3.03	0.003
	Age	−0.01	0.01	−2.08	0.038
	EER	<0.01	<0.01	0.48	0.632
	Time (hospitalization)	0.29	0.01	54.7	<0.001
5. %mBMI change	Intercept	87.37	2.80	31.25	<0.001
post-hospitalization	Age	−1.55	0.52	−2.97	0.004
	Calorie rate	<0.01	<0.01	−0.10	0.924
	Age	<0.01	0.01	−2.08	0.041
	EER	<0.01	<0.01	0.69	0.493
	Discharge calories	<0.01	<0.01	−1.67	0.099
	Age	<0.01	<0.01	1.49	0.141
	EER	<0.01	<0.01	−1.15	0.255
	Time (post-hospitalization)	0.08	0.11	0.70	0.489
6. Probability of re-	Intercept	−2.33	0.35	−6.65	<0.001
admission	Age	−0.19	0.17	−1.10	0.273
	Discharge %mBMI	0.05	0.04	1.05	0.296
	Age	−0.01	0.02	−0.53	0.597
	%mBMI rate	−1.50	1.67	−0.90	0.371
	Age	2.03	1.05	1.94	0.055
	Calorie rate	−0.01	0.01	−1.70	0.092
	Age	<0.01	<0.01	−0.75	0.452
	EER	<0.01	<0.01	−1.23	0.222

Time was scaled for hospitalization days or post-hospitalization days according to the specified model

*EER* estimated energy requirement, %*mBMI* percentage of median BMI

**Fig. 2:**
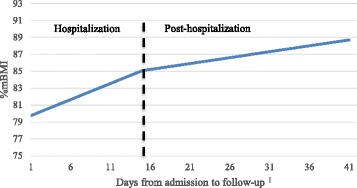
Changes in mean percentage of median BMI (%mBMI) during and after hospitalization. Figure 2 displays the results of the piecewise growth model illustrating the differing rates of change in %mBMI during and after hospitalization. ^1^The mean length of stay (14.98 days) and mean days between discharge and follow-up (26.07) were summed to determine the range of values for the x-axis. Follow-up data points represented only patients who completed this assessment

The piecewise growth model (Model 3) indicated that participants increased an average of 0.38 per day in %mBMI during hospitalization compared to 0.09 per day between the time of discharge and follow-up (Fig. 2), with both slopes being significantly greater than zero.

### Predictors of %mBMI

As indicated by results of conditional models [4, 5], younger participants evidenced lower initial %mBMI. Higher rates of caloric increases were associated with greater increases in %mBMI during but not after hospitalization (Models 4–5). The number of calories prescribed at discharge was not related to change in %mBMI post-hospitalization (Model 5). A Bernoulli model (Model 6) estimating the probability of re-hospitalization after discharge found that neither the rate of caloric change (OR: .99; CI: .98–1.00) nor rate of %mBMI change (OR: .22; CI: .01–6.06) was associated with re-hospitalization. Exploratory analyses revealed that the rate of caloric change was not correlated with the number of meal replacements administered (Spearman’s *ρ* =−.11, *p* = .345) or number of incidences of food refusal (*ρ* = .13, *p* = .492) during hospitalization.

Notably, three participants (2.33 %) evidenced decreases in %mBMI between admission and discharge. Compared to the rest of the sample, these individuals had significantly shorter lengths of stay (*M* = 1.89 days, *SD* = 1.03, *t*[120] =−15.04, *p* = <.001), lower calories (in relationship to their EER) prescribed at discharge (*t*[120] =−3.26, *p* = .001), yet the rate of caloric change did not differ significantly between groups (*t*[120] = .84, *p* = .491). Chart review of these cases found that these participants left prematurely against recommendations of the treatment team.

## Discussion

The present study aimed to assess the safety and effectiveness of a more accelerated refeeding protocol for hospitalized youth with AN and atypical AN. This protocol was largely consistent with the “start high, advance fast” approach [34], as evidenced in this sample by (1) a mean weight gain of approximately 1.4 kg per week; (2) medical stabilization for discharge within 15 days; and (3) a mean caloric increase of 164 kcal per day, which exceeded 3600 kcal by day 14. In addition, this protocol was able to be implemented with exclusively oral intake, with a small number of participants receiving NJ/NG tube feedings (*n* = 11) who were excluded from analyses.

Consistent with previous literature [16], youth with AN and atypical AN were able to tolerate more aggressive refeeding during hospitalization without any occurrences of refeeding syndrome. However, there was a significant risk of refeeding syndrome during hospitalization, as evidenced by hypophosphatemia in 47.3 % of participants. Nevertheless, while the proportion of participants who evidenced hypophosphatemia in this study was comparable to that observed in previous research [25], it appears more participants were prescribed phosphorus supplements in this study compared to others [25, 28]. It is important to note that electrolytes were monitored closely during treatment with aggressive phosphorus supplementation, which likely helped to prevent progression to refeeding syndrome.

In line with Golden and colleagues [26], hypophosphatemia was unrelated to caloric or %mBMI changes. Admission weight was not related to hypophosphatemia in this study, which is contrary to previous research demonstrating a relationship between the degree of malnutrition and hypophosphatemia [15, 45]. It is not clear why this was observed in the present sample, as participants evidenced similar admission %mBMI compared to other research [25]. One possible explanation is that preventative phosphorus supplementation for all patients who evidenced low or declining phosphorus values negated differences between patients with lower and higher %mBMI at admission. Furthermore, as noted by the recent position statement of the Society for Adolescent Health and Medicine [15], refeeding hypophosphatemia may present at any weight after a period of malnutrition. The present study also did not assess the degree of weight loss prior to hospitalization, which has been shown to be predictive of hypophosphatemia during treatment [26]. This may have occurred in the present sample, and it would be useful for future studies to explore this risk factor. Thus, while the current study did not demonstrate a relationship between hypophosphatemia and %mBMI at admission, given the prophylactic phosphorus supplementation, findings do not discount the need for medical providers to be cautious of the risk of hypophosphatemia in severely malnourished patients [15].

This was one of few studies to include a post-hospitalization assessment of a more aggressive refeeding protocol. Both caloric and %mBMI changes were greatest early on during hospitalization; not surprisingly, %mBMI change was greater during hospitalization compared to post-hospitalization. Nevertheless, participants continued to demonstrate significant increases in %mBMI after discharge, during which time they were engaged in outpatient therapies that supported continued weight gain. While previous studies have not specifically assessed the rate of caloric change, the present results suggested that higher rates of caloric increases were related to greater increases in %mBMI during but not after hospitalization. Thus, in addition to the existing support for starting at higher initial caloric levels [25, 26], these findings describe the specific relationship between rate of caloric change and degree of weight change during hospitalization. It is also notable that the rate of caloric change or number of calories prescribed at discharge were not predictive of post-hospitalization changes in %mBMI. Taken together, it may be that other factors (e.g., outpatient treatment) are more influential in the rate of weight gain post-hospitalization. Of note, both of the outpatient treatments (i.e., FBT and CBT-E) were structured evidenced-based protocols that have demonstrated effectiveness in facilitating weight gain in AN, and these specific treatments may have contributed to the observed post-hospitalization weight gain, which is consist with previous research [32]. Thus, it would be beneficial for future study to assess additional variables post-hospitalization that may facilitate early response and weight gain, which has been shown to predict long-term outcome in AN [46].

As hypothesized, higher rates of caloric and %mBMI increases were not related to an increased likelihood of re-hospitalization after discharge. These results replicate previous findings [25, 26], and may suggest that implementation of more accelerated refeeding protocols could result in shorter hospital stays and reduce cost and burden to patients and families. Furthermore, there is evidence to show that shorter lengths of stay followed by outpatient treatment are not detrimental to long-term physical or psychological outcomes in AN [32]. This is important to consider in light of the rising trend of residential treatments for eating disorders [47] and high cost of inpatient hospitalization [4, 48]. Brief inpatient medical stabilization in conjunction with evidence-based outpatient care may prove vastly more cost-effective and efficacious in outcome compared to prolonged inpatient and residential treatments, though further research is necessary to demonstrate these effects.

### Limitations

There were several limitations to this study. There was not a lower-calorie comparison group, which would have allowed for comparisons in %mBMI change and incidence of hypophosphatemia. The timing of caloric increases was not possible to assess, which would be helpful for future studies to address. The sample included only the first inpatient admission for youth with AN, and it is unclear whether this protocol would be similarly effective for other diagnoses, adults, or for more chronically ill patients. This study did not include measures of psychological symptoms that could assess changes in distress; however, it is notable there was not a significant relationship between caloric rate and meal replacements or food refusal, which suggests that higher caloric increases were tolerated. Re-admission beyond 4 weeks after discharge was not assessed; while there were no significant differences in many characteristics between those who completed the follow-up assessment and those who did not, it is unclear whether the latter group evidenced similar improvement in %mBMI after discharge. Participants who completed the follow-up assessment were also engaged in structured therapies, which makes it difficult to distinguish effects of the refeeding protocol compared to those of post-hospitalization treatment. Moreover, the types of post-hospitalization treatment (i.e., FBT vs. CBT-E) were not assessed as a potential moderators of post-hospital weight gain or re-admission, and future research is needed to assess whether these outcomes vary according to treatment type.

## Conclusions

Despite the aforementioned limitations, this study demonstrated youth with AN and atypical AN can tolerate more accelerated oral refeeding protocols without incurring refeeding syndrome. In particular, this study adds to the literature by describing the specific relationship between of the rate of caloric advances and weight changes during hospitalization. Although results lend preliminary support for more rapid caloric advances, additional research is necessary to assess long-term outcomes of such approaches, as well as investigate whether such approaches are feasible in settings outside the hospital. However, these results also underscore the importance of vigilant monitoring of indicators of refeeding syndrome, and treatment providers must be prepared to intervene with electrolyte correction. In sum, results suggest that the “starting higher, advancing faster” approach can possibly facilitate more cost-effective treatment, yet findings also underscore the importance of having available and accessible evidence-based outpatient treatments. Continued work is also needed to standardize the implementation of “starting higher, advancing faster” approaches for broader dissemination.

## Footnotes

1 Atypical AN was defined as %mBMI > 89 %. For participants for whom %mBMI could not be calculated because their age exceeded the maximum age specified in CDC BMI-for-age charts, atypical AN was defined as BMI >17.5.
